# Introducing *Jus ante Bellum* as a cosmopolitan approach to humanitarian intervention

**DOI:** 10.1177/1354066115607370

**Published:** 2015-10-14

**Authors:** Garrett Wallace Brown, Alexandra Bohm

**Affiliations:** University of Sheffield, UK; University of Sheffield, UK

**Keywords:** Cosmopolitanism, humanitarian intervention, human security, normative theory, Responsibility to Protect, theory and practice

## Abstract

Cosmopolitans often argue that the international community has a humanitarian responsibility to intervene militarily in order to protect vulnerable individuals from violent threats and to pursue the establishment of a condition of cosmopolitan justice based on the notion of a ‘global rule of law’. The purpose of this article is to argue that many of these cosmopolitan claims are incomplete and untenable on cosmopolitan grounds because they ignore the systemic and chronic structural factors that underwrite the root causes of these humanitarian threats. By way of examining cosmopolitan arguments for humanitarian military intervention and how systemic problems are further ignored in iterations of the Responsibility to Protect, this article suggests that many contemporary cosmopolitan arguments are guilty of focusing too narrowly on justifying a responsibility to respond to the symptoms of crisis versus demanding a similarly robust justification for a responsibility to alleviate persistent structural causes. Although this article recognizes that immediate principles of humanitarian intervention will, at times, be necessary, the article seeks to draw attention to what we are calling principles of *Jus ante Bellum* (right before war) and to stress that current cosmopolitan arguments about humanitarian intervention will remain insufficient without the incorporation of robust principles of distributive global justice that can provide secure foundations for a more thoroughgoing cosmopolitan condition of public right.

## Introduction

To make our argument for why principles of *Jus ante Bellum* are crucial to debates about humanitarian military intervention, the article is divided into four sections. The first section will undertake a brief survey of two moral arguments generally employed by cosmopolitans when justifying the use of humanitarian military intervention. This section will also highlight three persistently problematic questions that have remained largely unresolved within the cosmopolitan literature. From this, the second section explores three current themes within cosmopolitan debates about humanitarian intervention and how these themes intersect and potentially support our argument for the incorporation of principles of *Jus ante Bellum*. The third section seeks to illustrate that the lack of discussion about incorporating principles of *Jus ante Bellum* in debates about humanitarian military intervention is not simply confined to the realm of academia; *Jus ante Bellum* principles also relate directly to current preventative shortcomings within the Responsibility to Protect (RtP) and other international laws concerning the use of force. By exploring the language and practice of the RtP, it is possible to illustrate why it remains insufficient and morally malnourished on cosmopolitan grounds. Lastly, the fourth section will draw out three key implications of our argument for cosmopolitan thought more generally and how these relate to the practice of humanitarian military intervention. By exploring these implications, it will be argued that incorporating *Jus ante Bellum* principles into the cosmopolitan debate about the use of force will add greater consistency, legitimacy and focus to cosmopolitan humanitarian interventions and how our understanding of ‘intervention’ can better correspond to broader cosmopolitan ambitions.

Nevertheless, before moving forward, it is important to set and justify the parameters of this article. Primarily, although many non-cosmopolitans within Liberalism (Doyle, 2015; Pattison, 2010; Teson, 2003) and the English School (Booth, 2007; Dunne, 2013; Hurrell, 2003; Linklater, 2011; Wheeler, 2005), as well as advocates of the RtP (Bellamy, 2014; Evens, 2008), argue for humanitarian military intervention without also providing explicit links to *Jus ante Bellum* principles, a focus on cosmopolitanism has been maintained for the following reasons. First, as argued later, cosmopolitans often rely on strong deontological claims as a foundation for military intervention, which results in unique tensions that require more sophisticated justification for the use of violence than has been previously provided (Atack, 2005; Fabre, 2012; Reader, 2007). Second, cosmopolitans are the staunchest promoters of global justice within International Relations, yet they still insufficiently link their arguments for intervention to issues of distributive justice, or they have done so in a way that focuses on criminal justice (Archibugi, 2008; Brock, 2009; Fine, 2007; Held, 2010; Pattison, 2008) and cosmopolitan law enforcement (Hayden, 2005; Kaldor, 2003; Smith, 2007). Third, as cosmopolitans ourselves, we have remained uncomfortable, unsure and unconvinced by these existing cosmopolitan accounts supporting humanitarian military intervention and its possible cosmopolitan expression via the RtP (Held, 2010; Ossewaarde and Heyse, 2015; Sangha, 2012). As a response, this article represents an alternative and more comprehensive cosmopolitan vision, which we suggest can better legitimate cosmopolitan interventions as well as justify the ultimate aim of these interventions in the face of growing criticism. Lastly, although we recognize that the concept of *Jus ante Bellum* has clear heuristic links to *just war theory*, *human security* and *institutional cosmopolitanism*, due to limitations of space, a focus on cosmopolitan military intervention is maintained. The rationale is that engagement with these other traditions demands significant attention beyond a single article and that proper treatment is best suited for future research currently underway. That said, where germane, the article touches upon key interconnections as they relate to these traditions so as to highlight important implications and areas for future *Jus ante Bellum* research.

## Cosmopolitan humanitarian intervention and three persistent questions

When surveying the cosmopolitan literature, it becomes evident that a fair majority of cosmopolitans advocate the use of humanitarian military intervention as a means to respond to mass atrocity crimes or serious human rights violations. In arguing for humanitarian intervention, many cosmopolitans claim not only that there is a right to intervene, but also that those who are in a position to effectively respond have a duty to do so (Archibugi, 2008; Brock, 2009; Caney, 2005; Fabre, 2012; Fine, 2007; Hayden, 2005; Held, 2010; Kaldor, 2003; Pattison, 2008; Pogge, 1992; Sangha, 2012; Smith, 2007). The moral foundations underpinning this duty relate to two corresponding cosmopolitan principles.

First, cosmopolitans sustain a deontological commitment which suggests that all human beings have an intrinsic human worth and dignity that should not be violated. In opposition to consequentialism, human beings matter equally, and because humans have an equal intrinsic worth, it is not morally permissible to violate this worth. Furthermore, since this worth is held equally between all human beings, we have duties to come to the aid of other human beings so long as it does not at the same time greatly threaten our own ability to live lives worthy of what it means to be a human being. As all cosmopolitans argue, human dignity is universal in scope, so these duties apply globally to every human regardless of where they happen to reside and despite their cultural and political associations. Therefore, in terms of humanitarian intervention, since humans are the primary unit of equal moral concern, and since mass human rights violations threaten the basic dignity of other human beings (and/or ourselves), we have a moral duty to intervene.

Second, many cosmopolitans argue that humanitarian intervention is a justified mechanism to respond to large-scale injustices associated with human rights violations because when properly constituted, the intervention acts as a means to establish a condition of cosmopolitan public right. In this regard, many cosmopolitans often also see humanitarian intervention as a method of law enforcement by the international community (Archibugi, 2008; Fine, 2007; Hayden, 2005; Held, 2010; Kaldor, 2003, 2005; Smith, 2007) and/or as representing the fulfilment of a Kantian duty to transition *provisional rights* to a condition of *perfect rights* that are grounded in a more thoroughgoing condition of cosmopolitan law and constitutionalization (Roff, 2013). As Catherine Lu (2006: 135–136) summarizes, when a state:

fails to provide basic goods such as security, subsistence and justice within their borders, and when the domestic accountability systems are inadequate or incompetent, a cosmopolitan view of global order obligates the society of states, as well as the larger global civil society, to call sovereign power to account, and to intervene to alleviate the human suffering caused by the neglect, breakdown or abuse of sovereign power.

In this respect, intervention is seen as a juristic mechanism, which is grounded on deontological notions of human worth, and which can bring unstable political and legal orders in line with cosmopolitan political aspirations and values.

Nevertheless, despite the fact that a fair majority of cosmopolitans support the use of humanitarian intervention as a means to address gross injustices and the violation of human rights, there has been a relatively limited attempt to respond to the more problematic questions regarding the use of force often associated with intervention and the underlying cosmopolitan principles that justify its use. As Cecile Fabre (2012: 3–4) has recently pointed out in her more developed account of *Cosmopolitan War*, most cosmopolitan theorists ‘overlook the serious normative difficulties raised by military interventions which necessitate acts of killing’ and, as a result, ‘cosmopolitans … would do well to start thinking more deeply than they have done so far about war’.

The failure to address these questions sufficiently renders cosmopolitanism an underdeveloped theory of global cohabitation, which either cannot respond to the complexities of humanitarian military intervention or is unwilling to ‘own up’ to these unresolved tensions. In simple terms, the problems are obvious but remain unsettled, and the tensions stem from the fact that many cosmopolitans strongly advocate humanitarian military intervention and ‘cosmopolitan law enforcement’ as a means to save distant strangers, yet, at the same time, fail to provide thoroughgoing extrapolation for exactly why there are clear duties to intervene and why these duties can be consistent with the underwriting deontological principles of cosmopolitanism. In thinking about this, it is possible to find at least three questions that require a more thoroughgoing response by cosmopolitans who advocate a duty to intervene militarily on humanitarian grounds.

The first question relates to the nature of deontological arguments themselves and an inherent tension that becomes manifest when innocent life is destroyed as a result of military operations. The tension develops because in modern warfare, it is highly foreseeable, as well as nearly assured, that innocent people will die as a result of military intervention. Whereas strict utilitarian accounts can better justify any ‘collateral damage’ on the basis of meeting the terms of the ‘proportionality’ calculation and by fulfilling the requirements of ‘double effect’, any cosmopolitan deontological approach that strictly posits an intrinsic right over the consequential good will undoubtedly face the dilemma of demanding categorical duties to protect the dignity and rights of those beyond our borders while, at the same time, having to justify why those rights can be suspended in some cases. The problem being that if the deontological position suggests that ‘the right’ of human dignity should trump ‘the good’, then how can this right be suspended for the protection of the greater good? This is not to say that cosmopolitanism cannot reconcile this tension, but it is important to point out that cosmopolitan efforts to do so have so far been minimal and, in our opinion, incomplete.^1^

The second question relates directly to the preceding one, namely, if cosmopolitans argue for the deontological worth of human beings, and if military intervention will inevitably kill human beings (both innocent and belligerent), then can the cosmopolitan position only be consistent when adopting a pacifist position where any foreseeable destruction of human life remains absolutely impermissible? In this case, like the preceding one, the cosmopolitan has to defend why their position promotes peace and the deontological worth of human beings while, at the same time, advocating war and the known destruction of life as a means to bring about a cosmopolitan condition of peace (Reader, 2007). Although these questions lie at the heart of just war theory and are the focus of many debates within the literature on just war, cosmopolitans themselves have largely forgone any direct dealing with this difficult question.^2^

The third question faced by cosmopolitanism, which is the question we are focusing on in this article, relates to cosmopolitanism’s tight relationship to arguments for distributive global justice and how this body of work should link to cosmopolitan arguments for humanitarian military intervention. Specifically, when surveying the literature, it is unclear whether humanitarian military intervention simply represents a form of immediate criminal justice or whether the idea of ‘intervention’ is also to be fully incorporated into broader debates about distributive justice. Although Caney (2005: 225) does suggest that ‘an adequate normative account of global distributive justice cannot be divorced from an empirical account of war’, he discusses this only in a footnote, and it is unclear whether he believes that the reverse relationship also holds: that an adequate normative account of war cannot be divorced from an empirical account of distributive global justice and what we are suggesting are corresponding duties of *Jus ante Bellum*. This absence is indicative of the cosmopolitan literature more broadly, since discussions about cosmopolitan humanitarian military intervention have focused on the questions of ‘when, who and how’ (*Jus ad Bellum*, *Jus in Bello* and *Jus post Bellum*) without much reflection on the structural reasons ‘why’ the conditions for intervention persist in the first place (*Jus ante Bellum*).

When structural reasons are considered, they are usually discussed in relation to institutional debates about global authority mechanisms beyond the state and the necessary conditions for effective enforcement (Archibugi, 2008; Brock, 2009; Cabrera, 2010; Fine, 2007; Hayden, 2005; Kaldor, 2003; Pogge, 1992), versus addressing broader socio-economic structures that perpetuate conflict and the need for intervention in the first place. This is not to say that institutional cosmopolitanism is not underwritten by concerns for distributive global justice and global reform, since many cosmopolitans advocate poverty alleviation (Pogge, 2001), reforms to the United Nations Security Council (UNSC) (Archibugi, 2008; Brock, 2009; Hayden, 2005) and, in more radical cases, a need for a world government to fulfil the demands of global justice (Cabrera, 2010). Nevertheless, it is also fair to say that these institutional accounts have either assumed this connection or have not sufficiently and explicitly linked these socio-economic concerns to long-standing advocatory positions on cosmopolitan humanitarian intervention.

In other words, cosmopolitans focus mainly, if not exclusively, on the symptoms and aftermath of conflict rather than providing any detailed discussion about the underwriting causes of structural violence and how these relate to the demands of cosmopolitan distributive justice. As a partial response to this particular question (leaving the first two questions aside), we wish to argue two main points in relation to this particular shortcoming: first, that any consistent account of cosmopolitan humanitarian intervention must include *Jus ante Bellum* principles of distributive global justice in order for it to be fully consistent with broader cosmopolitan aims; and, second, that this is not simply an academic mental exercise, since the failure to address underlying structural causes associated with large-scale human rights violations is a clear weakness of the RtP, which has left it impoverished as both a normative and practical global constitutional device.

## Blurring the distinction between cosmopolitan criminal justice and distributive justice

There are three key intersections where what we are calling *Jus ante Bellum* overlaps with contemporary cosmopolitan discussions about humanitarian military intervention. However, before presenting these potential links, it is important to be clear about what we mean by *Jus ante Bellum*. As is typical in just war theory, Latin terms are often used to demark the various stages of war and the ‘just’ principles that must be satisfied before resorting to war (*Jus ad Bellum* — the right to war), when conducting war (*Jus in Bello* — right in war) and after the war (*Jus post Bellum* — right after war). In our use of *Jus ante Bellum* (right before war), we are suggesting two denotations.

The first, in line with Kant, is to understand the word *right* as having two corresponding meanings. One that refers to having an entitlement to act in the defence of others (or what Fabre calls the Hohfeldian transfer of rights) and another that refers to the underlying conditions of *public right* that must exist in order to fulfil *perfect rights* and/or the conditions of *publicity* necessary to move *imperfect rights* to *perfect rights*. In this last case, we are arguing that having the entitlement to act in defence of others must publically correspond to other conditions necessary for public right, in this case, a robust commitment to distributive principles that seek to eliminate gross inequalities that foreseeably lead to large-scale humanitarian crisis.

The second denotation relates directly to the ‘entitled’ use of force that follows from the first understanding just outlined. This suggests that if pro-intervention cosmopolitans are correct to claim that there is a strong duty to kill in order to save victims of direct violence, then there must also be a strong duty to prevent conflict from happening in the first place and that the fulfilment of this duty will require additionally robust commitments to global distributive justice. The logic underpinning this suggests that if ‘helping under a cosmopolitan view means providing the people affected with the means to exercise their own moral and social agency’ (Lu, 2006: 146), then this principle of assistance should surely also hold in relation to structural causes that make humanitarian military intervention necessary in the first place. As Newman (2009: 208) reflects, if human security and dignity is the ultimate goal of intervention, then this ‘suggests a duty to eradicate the conditions that create insecurity’. In this regard, *Jus ante Bellum* proposes that if we have duties to kill in order to save distant strangers from violence, then we also have duties to alleviate the suffering of distant strangers from structural conditions that have a significant probability of leading to large-scale crisis and conflict. As a result, not only should cosmopolitans care about immediate crisis, but, more importantly, cosmopolitans also need to be more explicit about the role that humanitarian intervention plays within a broadened cosmopolitan vision (and vice versa) — morally, institutionally, culturally and within the cosmopolitanization of international law.

One potential criticism of our focus on *Jus ante Bellum* is to suggest that the links between global structural socio-economic conditions and humanitarian crises are spurious and therefore lack the ‘relational conditions’ that any principles of cosmopolitan distributive justice will necessarily require (Miller, 2007: 23–50; Nagel, 2005: 114). As many critics of cosmopolitanism suggest, the global level does not empirically display the same level of ‘basic structures’ required for duties of justice to apply, and it is far more appropriate therefore to discuss humanitarian interventions as humanitarian assistance, which requires a lower threshold of duties than justice would demand.

In response, even if we agreed that principles of global justice only apply in relational conditions, this view seemingly ignores an increasing body of evidence which suggests that conditions of economic hardship, market inequalities and global poverty significantly increase the likelihood of conflict and mass killing. For example, Fearon and Laitin (2003) have shown that lower per capita income increases the likelihood of civil war. Similarly, Suzuki and Krause (2005) found that high levels of economic development reduced that likelihood. Furthermore, it is known that conditions of poverty increase the death rates associated with humanitarian violence by as much as a factor of 50 (Pogge, 2001). Cederman, Skrede Gleditsch and Buhaug (2013) also found that economic inequalities and market favouritism generated a high level of grievances in less stable political orders and, in turn, acted as one of the key motivators in mass conflict and civil war. Moreover, Mehta (2012) has shown not only that the way in which more developed countries purposely market and sell weapons in the developing world lacks the moral conscience of what we are calling *Jus ante Bellum*, but also that these sales significantly increase the death count in conflict areas where these weapons are ‘reasonably expected’ to be used on civilians or to affect non-combatants. Finally, there is increasing evidence to suggest that global market inequalities, economic disadvantage and poor control over natural resources result in increased instability and violence on a mass scale (Humphreys, 2003; Ostby, 2008: 144; Sobek, 2009: 175–189; Stewart, 2009). Although the causal relationship between these inequitable global economic structures and increased conflict is still clearly an area that requires more empirical investigation, on an intuitive level, it would seem sensible to suggest that a causal relationship exists and that poor or unfavourable economic conditions positively correspond to the increased violence generally associated with what Mary Kaldor (2005) calls ‘new wars’. In addition, there is also now a considerable body of empirical evidence to suggest strong links between current global economic systems and the perpetuation of abject poverty, and since poverty can be seen as a key driver of organized and disorganized violence (Newman, 2009: 208), it would seem that there are *prima facie* relational global conditions where *Jus ante Bellum* can be reasonably said to apply.

A second criticism is to suggest that what we are referring to as *Jus ante Bellum* is actually already covered within contemporary discussions regarding a justified war as ‘an option of last resort’ under the principles of *Jus ad Bellum*. In this way, the argument is that all other methods of avoiding conflict would need to be exhausted and that the various distributive measures we would argue for would be part of that effort.

Nevertheless, this is not what just war theorists are actually saying, and when surveying the literature, it is plain to see that the ‘option of last resort’ is directly in reference to an already-escalated humanitarian crisis and that the parameters for when to start measuring when a response is ‘of last resort’ relates to a situation well into an existing cycle of violence. As a result, the demands of *Jus ante Bellum* are more forward-looking than anything seemingly involved with *Jus ad Bellum* since we seek to expose deeper structural causes. As a result of this dissimilarity, we would suggest that, *prima facie*, a categorical difference between the two exists. In addition, the distinction we are making for *ante Bellum* duties to respond to structural factors is equally wanting within current iterations of the RtP (Secretary-General Report, 2014) and other documents on the use of force. As we will outline in more detail later, responsibilities to Pillar II are still framed as ‘assistance’ to respond to brewing crisis points, yet say near nothing about the role of external states in the perpetuation of entrenched economic and political structural causes that have a bearing upon crisis formation. As will be discussed later, the focus of the latest iteration of Pillar II requirements is still reactionary (Secretary-General Report, 2014), still vague on commitments and aimed at dampening existing state-based ‘hotspots’ versus reforming exogenous factors.

Cosmopolitans themselves could make a third critique by claiming that what we are calling principles of *Jus ante Bellum* are already implicit within any cosmopolitan approach to humanitarian intervention. In other words, a cosmopolitan could suggest that a commitment to global distributive justice is a given and that the advocacy for humanitarian intervention should be assumed as being couched within broader schemes of cosmopolitan justice.

However, there are two responses that can be made here. The first is to highlight that the connection between humanitarian military intervention and cosmopolitan justice is too important to leave as an implied relationship and that failing to fully embed humanitarian intervention in broader schemes of cosmopolitan justice creates greater ambiguities and misperceptions of the cosmopolitan project as a whole. These misperceptions can be witnessed in many of the reactions to cosmopolitanism from more critical voices who see cosmopolitanism as a potential form of Western cultural imperialism (Cohen, 2004; Douzinas, 2007; Hobson, 2012), as a form of biopolitics masked as humanism (Chandler, 2009) and/or as a form of capitalist exploitation (Harvey, 2009). As a result, if nothing else, the distinction of *Jus ante Bellum* seeks to try and make the connection between cosmopolitan justice and cosmopolitan criminal justice via intervention more explicit, not only for cosmopolitans themselves, but also for those critical of cosmopolitanism.

From this, a second response is to point out that one reason why cosmopolitans have failed to be explicit about the relationship between global justice and humanitarian intervention is because, all too often, they unnecessarily differentiate ideal and non-ideal theory as inhabiting different intellectual realms (à la Rawls). For example, the very first sentence of Caney’s (2005: 189) chapter on ‘Just War’ in *Justice Beyond Borders* claims that ‘thus far, this book has focused on ideal theory [Global Justice] … [now] I want to move from ideal theory to non-ideal theory … [because] a complete analysis must address what principles should apply when injustices have been committed’. The problem here is that this seemingly ignores the existence of a reverse relationship and, as a result, suggests that the structural injustices that have led to criminal injustices (crises) are not of immediate or equal importance and can be factored in separately. Furthermore, it is not exactly clear why responding to underlying structural drivers of injustice and the structural violence associated with the causes of humanitarian intervention is the sole purview of ideal theory whereas exploring just war principles is somehow non-ideally more ‘real’. Surely they are both very ‘real’ factors involved with crisis formation and relate to the empirical conditions to which normative theory must necessarily respond. Our argument here is not that cosmopolitans have categorically ‘got it wrong’, but to highlight that cosmopolitans are guilty of focusing too narrowly on justifying a responsibility to respond to the symptoms of crisis (as a non-ideal priority) versus demanding a similarly robust justification for a responsibility to alleviate persistent structural causes (as part of a larger non-ideal priority). If this is true, and if cosmopolitanism is going to provide a more thoroughgoing normative theory for global cohabitation, then this lacuna will have to be addressed more rigorously than has been done thus far.

Finally, *institutional cosmopolitans* might make three additional critiques of the *Jus ante Bellum* position presented here. First, like earlier, it could be argued that concern for global institutional and structural reforms already capture the spirit of *Jus ante Bellum* and thus render it redundant. Second, world government cosmopolitans could argue that the cosmopolitan demand for a condition of *perfect right* is only obtainable under a strong world authority with a correspondingly robust vertical dispersal of sovereignty. The implication is that the fulfilment of *Jus ante Bellum* would also be conditioned upon the existence of a world government (or close approximate) to enforce this condition, thus making *Jus ante Bellum* arguments essentially reducible to a world government position (Cabrera, 2010) and therefore redundant. Third, and related, *institutional cosmopolitans* might suggest that even if *Jus ante Bellum* does not essentially collapse into a world government position, it nevertheless would require robust institutional reforms at the global level and, as a result, is not as radical a cosmopolitan alternative as presented here.

In response, it is important to recognize that the argument for *Jus ante Bellum* is not offered here as a radical departure from cosmopolitanism, but as an alternative supplement to existing cosmopolitanism so as to sharpen and make the aims of the cosmopolitan project more explicit as a whole. As a result, the position argued here is not, necessarily, antithetical to institutional cosmopolitanism or world government arguments. This is because, theoretically, principles of *Jus ante Bellum* can remain agnostic in terms of institutional arrangements and can thus remain open to new institutional designs that go beyond existing accounts. In other words, maintaining a commitment to *Jus ante Bellum* does not commit one to world government, but only to institutional reforms that can meet its demands. Although this might, in the end, ultimately lead to something like a world government, it might not be a necessary condition, thus leaving scope for other pluralist accounts such as those found in global constitutionalism. Furthermore, by remaining institutionally agnostic, a commitment to *Jus ante Bellum* can better represent an intermediary position to advance cosmopolitan reform from existing practice, acting as a normative guide for how to think about humanitarian intervention. As will be illustrated in the third section, the application of *Jus ante Bellum* to the RtP reveals key legal and structural weaknesses, which allows for crucial normative judgements to be delivered.

To provide a more comprehensive cosmopolitan account, we think that there are at least three potential links where principles of *Jus ante Bellum* overlap with contemporary cosmopolitan discussions about humanitarian military intervention. First, under the banner of *Jus ad Bellum*, there is a requirement that the use of force is only justified when it is waged with the right intentions. Traditional justifications have usually claimed that a right intention can be: (1) to stop violence in order to establish a peace-keeping mission with the long-term aim of brokering a legitimate reformed government made up of warring factions; (2) to completely remove an unjust regime for the establishment of an externally imposed ‘just’ regime; and/or (3) to simply save distant strangers from immediate mass killing while leaving any long-term institutional solutions for debate during post-conflict reconstruction. Yet, underpinning this just war principle, particularly in relation to the justification for humanitarian military intervention, is the moral argument for the protection of human beings from harm. As mentioned earlier, in the case of cosmopolitan humanitarian military intervention, the grounding for the protection of individuals stems from their inherent moral worth and the equal dignity we owe them as fellow human beings. If this is the case, as all cosmopolitans suggest, then the right intention is not simply to stop the immediate violence (although this is certainly a principle of first intent), but to also establish a global condition of public right. In this regard, it would seem that for the cosmopolitan, a condition of ‘just intention’ must take into account the ‘just aims’ associated with those intentions and how those aims correspond to deeper structural socio-economic conditions that threaten to perpetuate violence. This is not just in regards to cases of immediate crisis, but also in relation to regions where there is a high potential for future crisis. Therefore, it would seem that for the cosmopolitan, having a ‘just intention’ is coupled with also having a ‘just aim’ that must necessarily go beyond the basic principles of *Jus post Bellum* as a way to incorporate cosmopolitan principles of distributive justice. If incorporated properly into a cosmopolitan humanitarian approach, this would include such activities as altering unjust economic conditions, curbing arms sales to conflict regimes (Oxfam, 2007; Pogge, 2001), limiting cash transfers to warring parties (Pogge, 2001), reforming unequal market conditions and trade relations (Nili, 2011), addressing systems of capital flight/profit shifting and strengthening poverty reduction efforts, and so on (IDRC, 2001). Although detailed here only briefly, the implications are considerable. Namely, ‘intention’ is always logically linked to ‘aim’, and any consistent cosmopolitan position therefore needs to make this link between their intention to save strangers and their concern for the broader conditions in which these strangers require saving in the first place, especially as measured against the bar and aims of cosmopolitan justice.

Another potential connection between *Jus ante Bellum* and cosmopolitan humanitarian intervention relates to questions regarding who is responsible to intervene. Specifically, as Fabre (2012: 181) has correctly pointed out in her book *Cosmopolitan War*:

there is another argument for the duty to intervene as grounded in considerations of reparative justice, whereby the IP [intervening party] is under a duty because it is in part responsible for the predicament in which TP’s [target parties] find themselves.

In other words, what Fabre is suggesting is that ‘whether a potential intervener is partly responsible for the rights violations to which the intervention is a response might be relevant to the assignment of the duty to intervene’ (Fabre, 2012: 189). In exploring this assignment of responsibility, Fabre uses the example of Belgium’s potential role in advancing the structural causes that ‘encouraged and fostered a climate of ethnic division and hatred’ (Fabre, 2012: 189) during its colonial mandate in Rwanda and how France furthered calamity by supplying weapons to the massacring parties right up to, as well as during, the Rwanda genocide. As Fabre states: ‘in such cases it might stand to reason that France had a primary reason to intervene’ (Fabre 2012: 189). Furthermore, in her own estimation regarding when such an intervention could be deemed successful, Fabre (2012: 190) claims that ‘humanitarian war will not successfully fulfill its just cause if it merely stops human rights violations in the short term: instead, it must secure the conditions under which the rights of its beneficiaries are secure in the long term’.

Nevertheless, this begs two questions. First, what if the responsibility for crisis is more related to structural inequalities and economic conditions built into the existing global order? Also, what if those structural conditions are, in some sense, understood to exist and to be perpetuated by certain powerful global actors? If responsibility should be assigned to those who protract these underlying causes, then under the existing arguments of global justice, many Western countries that have benefited greatly from the current unequal socio-economic structures that underwrite humanitarian crises would therefore be responsible for alleviating the effects of a crisis. Furthermore, if responsibility is associated with those who profit most from persistent economic inequalities, then responsibility could arguably move beyond Western countries to incorporate the Group of Twenty (G20), since this group represents roughly 90% of gross domestic product (GDP) output and is already responsible for 94% of official development assistance. In this way, demands for structural reform could be led by a small number of key actors. Second, if Fabre is right that successful intervention is related to long-term security, and if underlying global socio-economic conditions can be shown to have played a significant role in causing humanitarian crisis, then the question needs to be asked about the yardstick used to measure success and the key role that cosmopolitan theories of global justice should necessarily play in determining the long-term aims and successes of humanitarian intervention. In this way, what we are calling *Jus ante Bellum* plays two important normative roles: it provides a heuristic tool to help think about how to assign responsibility; and it helps us reflect more clearly about what conditions need to exist (or have existed that should not) in order to alleviate the underlying structural causes that help to perpetuate or escalate humanitarian violence in the first place.

Third, the idea of *Jus ante Bellum* has a potential connection to a number of conditions under the banners of *Jus ad Bellum* and *Jus post Bellum*. Although we cannot develop these in more detail here, *prima facie*, there are immediate connections and implications to be drawn out in terms of determining whether or not an intervention will ‘have a reasonable chance of success’, in relation to the ‘good outweighing the harms caused by military intervention’, and in relation to the interveners’ ‘responsibility to help reconstruct the vanquished country/countries’. In all of these cases, it would seem that cosmopolitans should have something meaningful to say in relation to what the demands of cosmopolitan justice require of these just war principles. That said, at the moment, of the few cosmopolitans who do engage with just war theory and humanitarian military intervention, the focus has largely been to justify why a cosmopolitan can endorse military intervention (Archibugi, 2004; Fine, 2007; Held, 2010; Kaldor, 2003; Pogge, 1992; Smith, 2007), to clarify in what cases an intervention is justified on cosmopolitan grounds (Brock, 2009; Fine, 2007; Pattison, 2008; Pogge, 1992) or to implant certain cosmopolitan values into existing just war clauses (Caney, 2005; Fabre, 2012; Hayden, 2005). What is underdeveloped is a revamped approach to cosmopolitan humanitarian intervention that fully integrates it within broader cosmopolitan concerns for global distributive justice. Otherwise, without this more thoroughgoing account, it is our belief that cosmopolitanism will remain largely an ‘add-on’ to the current debates without doing much to alter existing structures of unequal global constitutionalization, crisis prevention or the human suffering that is supposedly at the heart of why we have moral duties to intervene in the first place. Furthermore, a concern for what we are calling *Jus ante Bellum* is not purely an academic exercise because it is germane to contemporary international legal debates as they pertain to the use of force, and therefore has implications for how we think about the responsibility to protect distant strangers and what the demands of ‘prevention, reaction and rebuilding’ within the RtP should mean for cosmopolitans. It is to examining the RtP from this cosmopolitan perspective that we now turn our attention.

## The RtP as a response to symptoms not causes

So far, the argument has been that cosmopolitans have been focused too narrowly on the symptoms of crises without fully integrating cosmopolitan principles of distributive justice that could help mitigate the underlying causes that perpetuate humanitarian crisis in the first place. Nonetheless, as suggested in the previous section, this concern for *Jus ante Bellum* is also relevant to the ways in which we think about contemporary international law, as well as the persistent debates attached to the international society’s responsibility to protect distant strangers. Furthermore, there have been recent attempts by cosmopolitans to argue that the RtP embodies a cosmopolitan approach to intervention (Sangha, 2012) and that the RtP symbolizes a ‘cosmopolitan stepping stone’ within international law (Held, 2010; Ossewaarde and Heyse, 2015). Consequently, the aim of this section is to illustrate that the tensions that we suggest are engrained within the cosmopolitan treatment of humanitarian military intervention are also present in many aspects of the RtP.

### The RtP: Moving on from humanitarian intervention?

In 2001, the International Commission on Intervention and State Sovereignty (ICISS) produced its report on the idea of a ‘responsibility to protect’. Noting the increasing trend in international law towards protection of the individual, the RtP has sought to re-conceptualize the notion of sovereignty away from the traditional legal view of territorial control and towards a human rights-centred view of ‘sovereignty as responsibility’ (ICISS, 2001: 12, para. 2.8). The state retains primary sovereign responsibility for its citizens, but if the:

population is suffering serious harm, as a result of internal war, insurgency, repression or state failure, and the state in question is unable or unwilling to halt or avert it, the principle of non-intervention yields to the international responsibility to protect. (ICISS: xi)

In 2005, at the United Nations World Summit, the RtP was discussed widely and received a positive, if cautious, reception. This has been described by Nicolas Wheeler (2005: 97) as ‘190 states committed themselves to the principle that the rule of non-intervention was not sacrosanct’ in cases such as genocide. The RtP has not changed international law on the use of force, and the World Summit discussions stressed the need for states to undertake their responsibility to protect in line with international law, but it does represent affirmation of the UNSC’s view of certain human rights abuses as threats to international peace and security. Again, the latest iteration of the RtP in 2014 re-enumerates these commitments and, as will be discussed later, further seeks to outline ‘ways in which national, regional and international actors can assist States in fulfilling their responsibility to protect populations’ (Secretary-General Report, 2014: 2, para. 1). Thus, the UNSC and the ‘international community’ can be said to be increasingly accepting of humanitarian concerns as justification for military intervention, and accept military intervention as an appropriate response to humanitarian concerns. The RtP was invoked by the UNSC in its discussions on Libya, the Cote d’Ivoire and Mali, to name but a few situations, and also by the Human Rights Council. As such, it has been argued that the RtP is a coherent and useful ‘stepping stone’ in our responsibility towards vulnerable individuals and that it is gaining increasing legitimacy within international society (Ossewaarde and Heyse, 2015; Sangha, 2012).

The RtP has attempted to be more than just a doctrine on humanitarian intervention and providing *military* help to solve human rights abuses. It is intended to provide a more complete account of our responsibility towards vulnerable individuals, with a wider focus than simply *Jus ad Bellum* questions of whether/when to undertake humanitarian military intervention, including the prevention of crises and post-conflict rebuilding. For example, the ICISS (2001: 20, para. 3.9) report commented that conflict prevention was key, highlighting the importance of early warning of conflict hot spots. The ICISS (2001: 19, para. 3.2) report also noted the responsibility of the international community to fulfil its secondary responsibility towards vulnerable populations using economic, political and legal, as well as military, means. In addition, the latest report of the Secretary-General in 2014 outlines Pillar II pledges stipulating that the international community will ‘support the United Nations in establishing an early warning capability, and assist those which are under stress before crisis and conflicts break out’ (Secretary-General Report, 2014: 2, para. 1).

This wider approach to human rights abuse prevention is still based on the assumptions of Fabre and others: ‘a political regime has a claim to govern over a given territory only if it respects and protects the fundamental rights of its individual members’, if it fails to do so, then ‘a cosmopolitan ethics of assistance yield a duty to provide military help to those in need’ (Fabre, 2012: 170, 207). Despite arguments that the RtP is fundamentally different from the previous concept of humanitarian intervention, it will be shown that the RtP suffers from the same misconceptions about duties towards distant strangers that have consistently haunted the humanitarian intervention debate. The key problem with the RtP’s understanding of global injustice, explained in more detail in the next section, is that it is insufficiently cosmopolitan because the RtP locates the blame for crises with the government of the state that is perceived to be unable or unwilling to protect its population, providing for the ‘international community’ of willing states to exercise a secondary cosmopolitan responsibility towards distant strangers. In addition, the Pillar II commitments of the RtP as outlined in 2014 continue to posit a language of ‘assistance’ versus ‘duties’ to reform unjust exogenous structural causes. In constructing the problem and the solution in this way, the RtP perpetuates the same mistakes of the humanitarian intervention debate, overlooking the international community’s involvement in the intrinsic injustices operating at the systemic level, which themselves contribute to the outbreaks of violence to which the RtP seeks to respond.

### The RtP: New doctrine, same mistakes

The ICISS (2001: 19, para. 3.2) report expresses the view that a ‘firm national commitment to ensuring fair treatment and fair opportunities for all citizens provides a solid basis for conflict prevention’. If this national commitment is not forthcoming, there is a role for the international community in identifying local conflict triggers (ICISS, 2001: 19, para. 3.4) and putting pressure on the national government to institute fair national structures of good governance, whether bilaterally or through international financial institutions (ICISS, 2001: 27, para. 3.41). These base commitments were reiterated in 2014: the international community should provide ‘encouragement, capacity-building and protection assistance, and good examples of good national, regional and international practice’ (Secretary-General Report, 2014: 1). If these preventative efforts fail, then the international community still has a further responsibility — that of reaction rather than prevention. This reaction does not have to be military in nature, moving away from humanitarian intervention’s focus on the use of force and including political, legal and economic measures such as sanctions or diplomacy (ICISS, 2001: 29, para. 4.5). The international community also has a responsibility to rebuild society if destructive force is used, with an emphasis on ‘local ownership’ of this process, together with a national commitment to fair treatment and good governance that was held to be key at the prevention stage (ICISS, 2001: 39, para. 5.2; Secretary-General Report, 2014: 11, paras 41–42). ICISS focuses on national structures and responsibilities, in particular, within the pillars of prevention (Pillar II) and reaction (Pillar III) (understandably, since the responsibility to react must necessarily be from the international community if the national level has failed in its responsibility). It is within these pillars that the state as provider of domestic justice is most evident — justice in the RtP being equated with good governance, itself understood in terms of democratic representation, fair treatment and fair opportunities for individuals.

It can thus be seen that the RtP’s focus is on national commitments by a government towards its citizens, with the international responsibility focused on curing defects at the national level and building a good society through, for example, international financial institutions’ good governance efforts or through post-conflict rebuilding. This view of the causes of injustice and the way to achieve justice suffers from several shortcomings, which are also mirrored in the cosmopolitan approach to humanitarian military intervention, as discussed in the second section. As we have argued, considerations of *Jus ante Bellum* are not only a necessary component to any cosmopolitan approach to humanitarian intervention, but also an important factor to take seriously in current debates surrounding the RtP.

One immediate problem with the RtP’s approach to humanitarian crises is that it views the state as the only appropriate entity that can deliver social justice, as well as the only source responsible for the underlying problems associated with humanitarian crisis. Although the strengthening capacities of the private sector and development aid strategies are briefly mentioned in the latest iteration of Pillar II commitments as means to assist crisis states, they are framed as ‘assistance’ mechanisms and not as potential causal factors that require immediate reform as a demand of justice. As a result, the ‘real’ causes of threats to individuals are perceived as stemming near exclusively from the local level. This presentation of the causes of threats and their potential solutions fails to accept the possibility that the international community has contributed to the conditions of injustice from which it then wishes to rescue people. These conditions of injustice for which the international community is arguably responsible include both immediate contributions to civil and political strife in problem states and more general, systemic problems of socio-economic poverty and inequality. In the second section of this article, we suggested a potentially significant link between socio-economic inequality and outbreaks of violence in which atrocities occur. It is therefore problematic that the RtP does not fully take account of this link in its supposedly new Pillar II approach to the idea of humanitarian military intervention.

Hilary Charlesworth (2002), mirroring many of our concerns, views the focus on crises (and their consequent need for urgent military action in response) as also being problematic in contemporary legal debates. She comments that ‘using crises as the focus means that the “fundamental” questions and enquiries are very restricted’ and argues that this focus ‘diverts attention from structural issues of global justice’ (Charlesworth, 2002: 377, 382). We have already argued, in the second section, that these structural issues (such as global poverty and health inequality) are important not only in their own right as issues of justice with which cosmopolitans should be concerned, but also because such structural problems are closely related to outbreaks of violence. By framing the key problem facing the international community as four particular acts of mass atrocity, the RtP perpetuates this crisis focus and neglects the importance of socio-economic issues and their significant relationship with the violence to which the RtP itself seeks to respond. A well-used example can be taken from some 1998 statistics. In this year, there were 588,000 deaths resulting from war and 736,000 resulting from social violence; during the same period, starvation claimed the lives of 18 million people, 34 million people worldwide were suffering from AIDS and HIV, and 1.2 billion people were suffering from malnourishment (Hurrell, 2003: 42). Even if particular conflicts can be shown to result in higher death tolls than the violence in 1998 (e.g. 800,000 died in Rwanda and over 200,000 are thought to have died in Syria), these statistics still give pause for thought. The disparity between concern for civil and political deaths and socio-economic deaths is troubling in its own right, purely ‘on the numbers’. However, it is also troubling from the perspective of the consistency of the cosmopolitan argument in favour of helping strangers.

Related to this crisis focus, a second problem with the RtP is that it poses the question of intervention — or any response under the ‘reaction’ pillar (Pillar III) — as simply one of ‘doing something or doing nothing’ (Chesterman, 2000: 108) — of being in favour of humanitarian military intervention or of adhering to a wholly unacceptable ethical theory that allows wholesale slaughter (Teson, 2003: 93). As Glennon (1999: 5) says: ‘[a] child saved from ethnic cleansing in Kosovo by NATO’s [North Atlantic Treaty Organization’s] intervention is no less alive because the intervention was impromptu rather than part of a formal [legal] system’. Yet, this debate poses the issue of humanitarian intervention in terms of the opposition between the rules of international law on non-intervention and non-use of force^3^ versus the failure of some governments to obey international human rights norms towards their own citizens (Teson, 2003; Weiss, 2007: 12). Although the RtP was intended to move on from the humanitarian intervention debate (e.g. by focusing on victims rather than interveners), debates surrounding RtP continue to return to the idea that a crisis has arisen, through national failure or fault, and the international community — a neutral entity or a force for good — has the option of acting successfully to deal with the problem. Again as suggested in the second section, the international community is, in fact, already deeply involved in non-military interventions in many problem states, through complex economic and political relationships (whether transnational corporations or international institutions). This calls into question the ‘neutral’ starting point of doing something or doing nothing in response to a crisis — the international community is already ‘doing something’ in many states in which problems develop (Alston, 1992: 107; Bellamy, 2003: 329; 2009: 99, 131). It thus seems that the debate has not moved forward as much as RtP supporters hope. Thus, despite the RtP’s attempt at providing a broader, more nuanced response to the issues raised by mass atrocities in the 2014 report of the Secretary-General, the issue nevertheless returns consistently to the issue of *international* military intervention as a reaction to *national* failures, with little consideration of how the international community is itself morally and legally implicated in promoting various causal factors that help lead to injustice and violence.

Drawing upon this binary representation of the possible responses to crises by the international community, a third problem with RtP relates to how the question of justice is framed and its focus on a society of ‘good’ states and the manifest failure of a particular state. While intending to move past the human rights–non-intervention deadlock of humanitarian intervention, the RtP falls prey to this same deadlock and division between good and bad states through its location of blame for the main problems faced by vulnerable populations (civil wars, repression and state collapse). This blame is located at the national level, from a national governmental failure to ensure fair treatment (Gordenker and Weiss, 1993; Reisman, 1991; Teson, 1995, 1996). The RtP perceives the international community’s preventative role as being in ‘assisting’ to identify local triggers of conflict (and responding to these triggers with political, legal, economic and military measures) rather than in changing global structures that perpetuate potential conflicts (ICISS, 2001: 19, paras 3.3, 3.4).

In addition to the general points made by Alston, Bellamy and others, we use a more specific example to demonstrate our point. The crisis that developed in the Balkans in the early 1990s was suggested to be the result of long-brewing ethnic hatreds and tensions — local problems of local people. Compared to this local cause of problems, it was natural to view the international ‘level’ of actors as being able to come to the rescue — rather than viewing international actors as potentially part of the local causes (Orford, 1997: 444). Nevertheless, examining this local–international division more closely, Anne Orford (1997: 453) and Susan Woodward (1995: 3) both find a chain of causation leading from the international community’s activities to the local instability and devastation that took place in the region. The different Yugoslavian provinces had coexisted fairly peacefully and autonomously from the central government and from each other, with limited calls for independence and secession. The World Bank and International Monetary Fund (IMF) then attached stringent conditions to Yugoslavia’s repayment of its Cold War debts. These included changes to the constitution that increased centralized control and reduced the independence of the country’s autonomous regions. As well as this general decrease in self-determination at the province level, the specific changes introduced by this newly powerful central government (at the behest of the international institutions) included reduced state education availability and reduced labour laws relating to worker protection. Changes such as these resulted in increased overall poverty and unemployment, and increased insecurity and social exclusion — together with calls for independence of various regions so that these locales could then reverse the damage done by the social changes forced upon the country by the central government and international actors (Orford, 1997: 454). Similar points can be made in relation to the international–local relationships in Rwanda — both Belgium’s colonial policies and, later, neoliberal economic policies enacted in Rwanda’s coffee sector. These helped to create conditions of structural tension that played a role in the genocide (Bohm, 2013: 247; Jones, 1995; Mamdani, 2001: 13; Robbins, 2002).

What this point highlights, in connection to our previous argument, is that it is highly conceivable that the international system is involved in perpetuating causes of conflict. Although good development aid practices and private sector compliance with the *2011 United Nations* *Guiding Principles on Business and Human Rights* are cited as ways to reduce ‘risk’ within Pillar II refinements (Secretary-General Report, 2014: 7, para. 26), these continue to be under-defined in terms of duties of justice, and as the UN itself admits, ‘there is still too little will to operationalize prevention’ (Secretary-General Report, 2014: 18, para. 73). Of course, the Balkan crisis pre-dates ICISS and the RtP, but the same problems can be seen in the RtP era: the RtP locates the problem of mass atrocities, and its causes, firmly at the local level — and correspondingly places the solution to this problem in the hands of ‘enlightened’ states in the international community (Glennon, 1999: 3). For example, despite Orford’s suggestion that the interference of the ‘international community’ — particularly in the form of the World Bank and IMF — was a significant contributing factor to the violence in the Balkans, the RtP views these institutions as part of the international community’s secondary responsibility to protect people by encouraging good governance and human rights. This more nuanced account of the causes of local problems challenges the unidirectional focus of both the RtP and supporters of humanitarian military intervention such as Fernando Teson (2003) — this unidirectional focus being evident in the idea that the universal nature of human rights obligations requires us to rescue those in danger of atrocities. Surely such universal obligations should be engaged before the idea of ‘rescue’ ever becomes necessary — in a universal obligation not to contribute to the injustices and crises from which we then wish to rescue people. Of course, those individuals actually carrying out acts of genocide or ethnic cleansing are not blameless — but the government of the problem state, and the individuals within the state in question, are not the *only* cause of harm. Although Weiss (2007) argues that in the ICISS consultations, nobody asked for less intervention, they often wanted more, this argument (that intervention is what the victims want) does not respond adequately to the question of why military intervention is ‘required’ in the first place.

That said, one paragraph of the RtP does give some acknowledgement to the elements associated with what we are labelling *Jus ante Bellum* by outlining some ‘root’ causes of conflict — those of ‘poverty, political repression, and uneven distribution of resources’ (ICISS, 2001: 22, para. 3.19). This is also mirrored in the *Report on State Responsibility and Prevention* (United Nations, 2013: 1), where armed conflict flows from ‘persistent patterns of discriminations; economic deprivation and related disparities; weakness in state structures; motives or incentives to commit atrocity crimes, including the presence of crimes’. ICISS also criticizes the level of Cold War debts and the trade policies of richer countries, both of which prevent poor countries from dealing with poverty and underdevelopment (ICISS, 2001: 20, para. 3.8). However, despite this acknowledgement, the question of the potential responsibility of the international community for this poverty (and therefore the associated violence escalation (Galtung, 1996)) is sidestepped. This is because the RtP posits that root causes, such as poverty, can be resolved by democratic participation and the strengthening of human rights at the *national* level (ICISS, 2001: 19, para. 3.2). This means that the RtP avoids considering the extent of the international community’s duty towards non-citizens in relation to poverty and inequality in a thoroughgoing manner — it is not at all clear what responsibility external states have towards rectifying their role in perpetuating these root causes and ICISS devotes a mere six lines (from 108 pages) to the issue of how schemes of distributive justice could help produce international order (ICISS, 2001: 23, para. 3.22). Economic development is seen more as part of *Jus post Bellum*, in the international community’s duty to rebuild and encourage growth, than any concept of *Jus ante Bellum*. In other words, like most cosmopolitans writing on intervention, the RtP does not sufficiently address the global structural causes that have played a significant part in creating the conditions associated with conflict escalation. As has been noted by scholars of the RtP, ‘the preventive dimension … has been consistently side-lined’ (Sharma, 2010: 127) and was seemingly only ‘tagged on in order to make military intervention more palatable’ (Bellamy, 2011: 19).

Most principles of justice are deemed to end at state boundaries — the state must treat its people justly, through equality of political rights and opportunities (Rawls, 1971). Other states are not required to treat other individuals — non-citizens — justly, by increasing duties to non-citizens in the socio-economic realm. As most just war principles of social justice end at state borders (and are, in any event, focused more on civil and political than socio-economic rights), international legal regimes governing fair access to food or drugs are not seen as a problem relating to international order with which the international community should concern itself. A wider emphasis on socio-economic rights (and a wider emphasis on responsibility beyond the national, domestic level of a government and its citizens) as part of ideas of duties of justice would consider international responsibility to individuals across the globe — both for global socio-economic inequalities and specific outbreaks of violence. By neglecting this important issue, the RtP has not, in fact, ‘moved on’ from the humanitarian intervention debate (and the problems that dog the cosmopolitan argument in favour of humanitarian military intervention) in any significant way.

As a result, this raises important questions about ethical theory and its relationship to international law, and why it is possible to prohibit the abuse of certain civil and political rights by a government but not prohibit the abuse of socio-economic rights affected by global structures. It seems inconsistent for a theory of ethics (which underpins the RtP and attempts to move international law ‘forward’ in human protection) to prohibit certain forms of intra-state violence but fail to prohibit prior interference from other states or institutions that can lead to instability, poverty and the prohibited acts of violence themselves.

To respond, we argue that both cosmopolitans and advocates of RtP should rethink the role of ‘sovereignty as responsibility’ away from placing blame for crises only with the government of the state in which the crisis occurs. This is because if cosmopolitans are truly concerned with protecting individuals from harm, and the harms directly related to military crisis, then fulfilling these aims actually requires a more nuanced approach to the relationship between mass atrocities and global poverty and inequality. If this is to be done thoroughly, then we believe that this will necessarily require a better connection to be made between cosmopolitan justice and current arguments for intervention, as posed by the RtP.

## Conclusion: *Jus ante Bellum* and the demands of the consistent argument within cosmopolitanism and its approach to humanitarian military intervention

In this article, we have argued that many cosmopolitan claims about humanitarian military intervention are incomplete on cosmopolitan grounds because they ignore the systemic and chronic structural factors that underwrite the root causes of these humanitarian threats. By way of examining cosmopolitan arguments for humanitarian military intervention and how systemic problems are further ignored in international legal tenets such as the RtP, we have suggested that many contemporary cosmopolitans are guilty of focusing too narrowly on justifying a responsibility to respond to the symptoms of crisis versus demanding a similarly robust justification for a responsibility to alleviate persistent structural causes. Although we fully recognize that immediate principles of humanitarian intervention will, at times, be necessary, the purpose of this article has been to draw attention to what we are calling principles of *Jus ante Bellum* (right before war) and to stress that current cosmopolitan arguments about humanitarian intervention will remain insufficient without the incorporation of robust principles of distributive global justice that can provide secure foundations for a more thoroughgoing cosmopolitan condition of public right. In making a stronger link between a cosmopolitan approach to humanitarian intervention and cosmopolitan principles of global distributive justice, three positive implications are generated.

First, fully embedding arguments for humanitarian interventions and the demands thereof into robust principles of cosmopolitan justice will help sharpen the aims of intervention, as well as generating a better philosophical response to those who view cosmopolitanism as a form of imperialism. As argued earlier, linking intervention to an idea of *Jus ante Bellum* will help to clarify the motivating aims behind ‘just intent’ under the banner of *Jus ad Bellum*, as well as solidifying the chances of greater success for the intervention itself.

Second, fully embedding arguments for humanitarian interventions and the demands thereof into robust principles of cosmopolitan justice will help to create a greater sense of legitimate authority for the interveners. This is because, if taken seriously, principles of *Jus ante Bellum* would have already required considerable efforts by the international community to correct structural elements that were greatly affecting stability. Again, if done properly and with a mutually consistent sense of global justice, the position of legitimacy held by the international community (or those intervening) would be heightened. This supports not only the legitimate authority clause of *Jus ad Bellum*, but also the idea of a legitimate ‘international society’, which can only assist in making any necessary intervention less bloody and more widely accepted because of its grounding in a more thoroughgoing notion of ‘justice’.

Third, we suggest that there is a strong element of consistency attached to the principle of *Jus ante Bellum*. Namely, it seems completely incoherent to claim that states owe a universal duty of justice and protection of human rights to their citizens while also claiming that there are no universal duties to people beyond borders until violence erupts. What is unclear in this case is why physical violence demands a corrective duty, yet other forms of known structural violence do not. Furthermore, intuitively, it seems incoherent to claim that there is a duty to kill in order to save distant strangers, but not a duty to alter unjust structural conditions that will foster that need to kill in the first place. In this regard, fully embedding arguments for humanitarian intervention and the demands thereof into robust principles of cosmopolitan justice can only help to create a more consistent argument by cosmopolitans. If this makes sense, then any policy such as the RtP, which seeks to protect vulnerable individuals from harm, requires a change of priorities away from a focus on military interventions into crisis situations, and towards redressing structural, systemic causes of crises before they occur. In a modern context, this will, of course, include health, employment opportunities and education. Importantly, it will also include the seemingly obvious, but so far avoided, restriction upon arms sales and unethical corporate activities in unstable regions, together with a commitment to non-military, consensual diplomatic peace processes.

## Notes

1. Both Cecile Fabre (2012) and Simon Caney (2005) have attempted to reconcile this tension. In both cases, the argument is made that a middle position between deontological and utilitarian accounts can be found via what they call a ‘rights-centred’ approach. Stronger links to *Jus ante Bellum* strengthen this middle position and better articulate the aims of cosmopolitan intervention.

2. Fabre (2012: 13–14) has also given some attention to this problem, conceding that there is an inherent deontological tension in cosmopolitanism as it relates to the use of force, but that for practical purposes, cosmopolitanism can be adapted, and that cosmopolitans should not resist the urge to protect global goods to which we all have claims as human beings. As a result, where there are clear threatening cases, military intervention is justified. In our opinion, stronger links to *Jus ante Bellum* strengthen this cosmopolitan appeal to ‘global goods’ and thus supplement and support a more comprehensive cosmopolitan account, as advocated by Fabre.

3. Art 2(7) and 2(4), UN Charter.
